# Supplementary material for: Dynamic coordination engineering of 2D PhenPtCl2 nanosheets for superior hydrogen evolution
Source: Nat Commun. 2024 Jan 9;15:385. doi: 10.1038/s41467-024-44717-1 (PMC10776781; doi:10.1038/s41467-024-44717-1)
Supplement: Supplementary file 1 — Supplementary Information [file 41467_2024_44717_MOESM1_ESM.pdf]

Supplementary Information for

Dynamic coordination engineering of 2D $PhenPtCl_2$ nanosheets for superior hydrogen evolution

Gonglei Shao[1,#,*], Changfei Jing[2,3,#], Zhinan Ma[4], Yuanyuan Li[5], Weiqi Dang[6], Dong Guo[1], Manman Wu[1], Song Liu[7], Xu Zhang[1], Kun He[2], Yifei Yuan[2], Jun Luo[8], Sheng Dai[3,*], Jie Xu[2,*], Zhen Zhou[1,*]

[1] Interdisciplinary Research Center for Sustainable Energy Science and Engineering (IRC4SE[2]), School of Chemical Engineering, Zhengzhou University, Zhengzhou 450001, P. R. China

[2] College of Chemistry and Materials Engineering, Wenzhou University, Wenzhou 325035, P. R. China

[3] Feringa Nobel Prize Scientist Joint Research Centre, School of Chemistry and Molecular Engineering, East China University of Science & Technology, Shanghai 200237, P. R. China

[4] School of Chemistry and Chemical Engineering, North University of China, Taiyuan, Shanxi 030051, P. R. China

[5] School of Sciences, Henan University of Technology, Zhengzhou, 450001, P. R. China

[6] National Laboratory of Solid State Microstructures, School of Physics, Collaborative Innovation Center of Advanced Microstructures, Nanjing University, Nanjing 210093, P. R. China

[7] Institute of Chemical Biology and Nanomedicine (ICBN), State Key Laboratory of Chemo/Biosensing and Chemometrics, College of Chemistry and Chemical Engineering, Hunan University, Changsha 410082, P. R. China

[8] ShenSi Lab, Shenzhen Institute for Advanced Study, University of Electronic Science and Technology of China, Longhua District, Shenzhen 518110, P. R. China

[#] Both authors contributed equally.

* Correspondence to: shaogonglei@zzu.edu.cn; shengdai@ecust.edu.cn; jiexu@wzu.edu.cn; zhouzhen@nankai.edu.cn

Supplementary Note 1: Morphology of 2D PhenPtCl_2 nanosheets

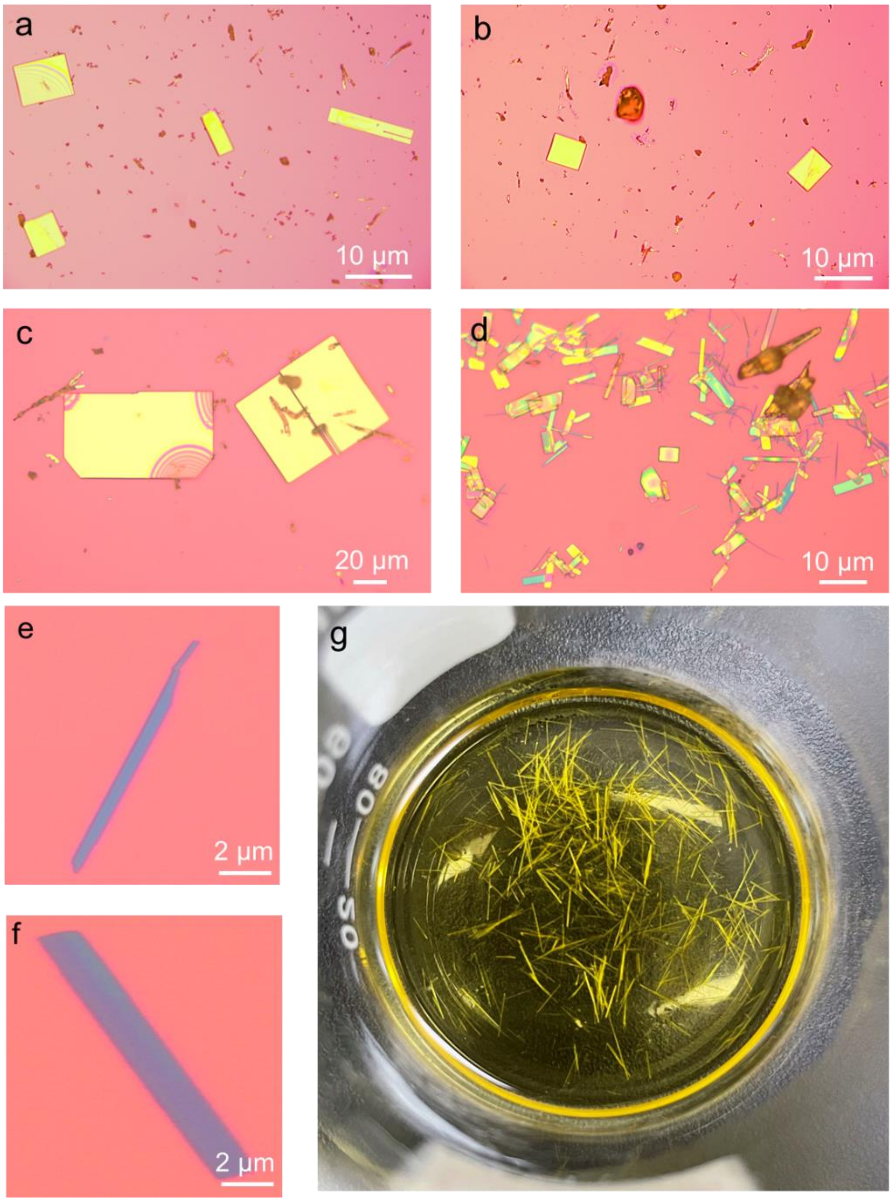

Supplementary Fig. 1. Morphology of 2D PhenPtCl_2 nanosheets. (a-f) Optical images of 2D PhenPtCl_2 nanosheets at 25 °C. (g) Camera photos images of recrystallization for PhenPtCl_2 nanosheets at 25 °C. From the optical images, the 2D PhenPtCl_2 crystals exhibit an obvious lamellar structure and a well-defined crystal morphology. Moreover, the size of 2D PhenPtCl_2 crystals range from a few microns to hundreds of microns. In the recrystallized solution, the 2D PhenPtCl_2 crystals show a yellow elongated crystal structure, and the size of 2D PhenPtCl_2 crystals can grow to more than 1 cm, which provides a good foundation for the characterization of single crystal structure.

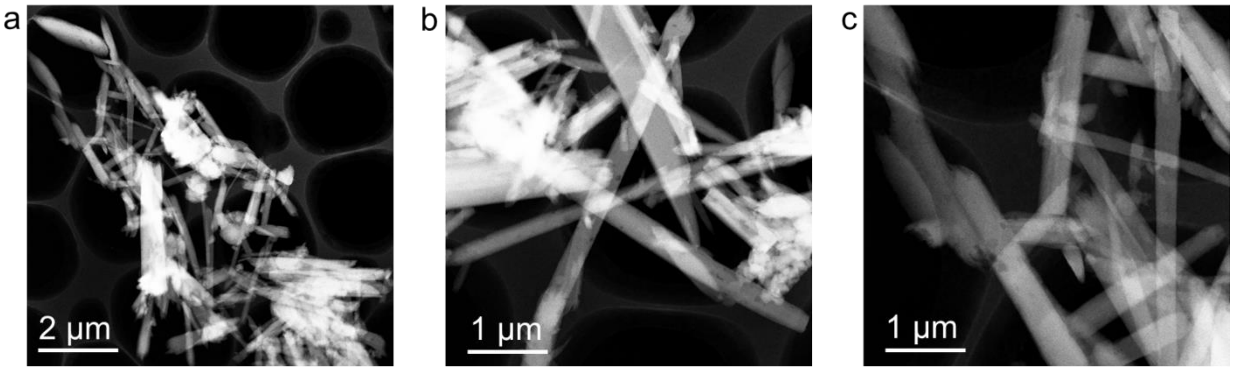

Supplementary Fig. 2. (a-c) Low-resolution STEM images of 2D $PhenPtCl_2$ nanosheets at 25 °C. These low-resolution STEM images reveal a lamellar structure and relatively uniform in thickness.

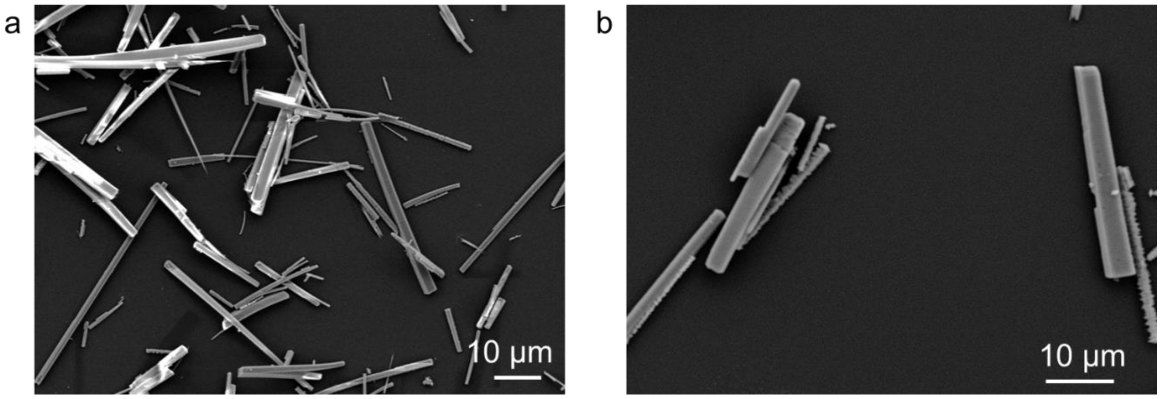

Supplementary Fig. 3. SEM images of 2D $PhenPtCl_2$ nanosheets at (a) 25 °C and (b) 100 °C. These SEM images of 2D $PhenPtCl_2$ nanosheets at 25 °C and 100 °C all show the obvious elongated band structure and the relative perfect crystallinity.

Supplementary Note 2: Structural analysis of 2D $PhenPtCl_2$ nanosheets

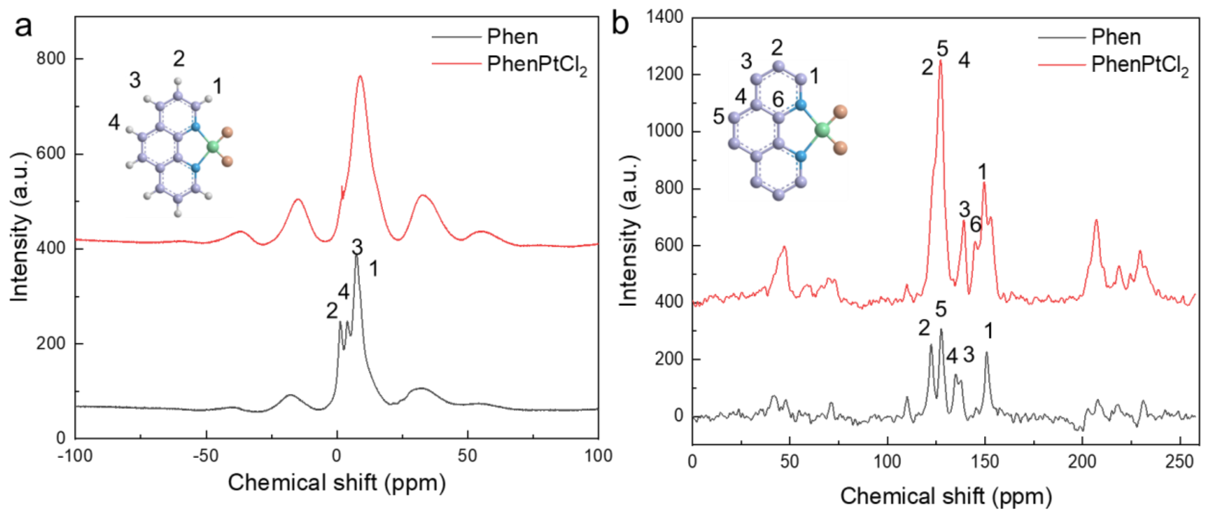

Supplementary Fig. 4. (a) H spectrum and (b) C spectrum of the solid state nuclear magnetic resonance for 2D $PhenPtCl_2$ organic crystals at 25 °C. Relative to Phen organic crystals, the H and C spectra of 2D $PhenPtCl_2$ organic crystals are only slightly shifted, which is caused by the Pt metal atom.

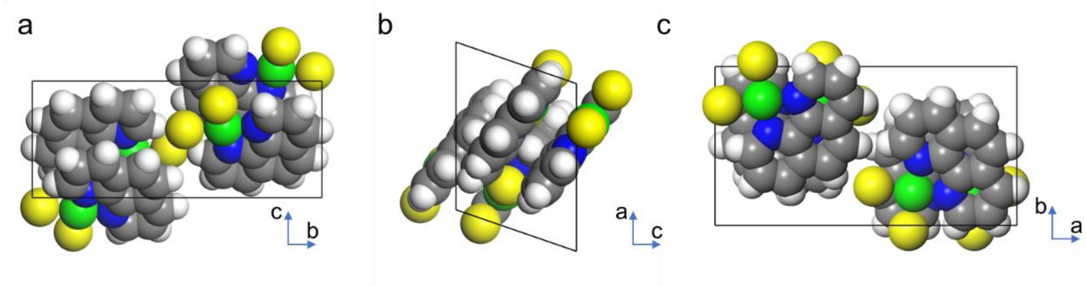

Supplementary Fig. 5. Atomic structure of 2D $PhenPtCl_2$ from (a) a, (b) b and (c) c crystal axis directions. Yellow, green, grey, blue and white atoms represent Cl, Pt, C, N and H atom, respectively. The layered arrangement of 2D $PhenPtCl_2$ crystal can be seen from the a, b and c crystal axis directions.

Supplementary Note 3: Atomic structure characterization of 2D $PhenPtCl_2$ nanosheets at 25 °C

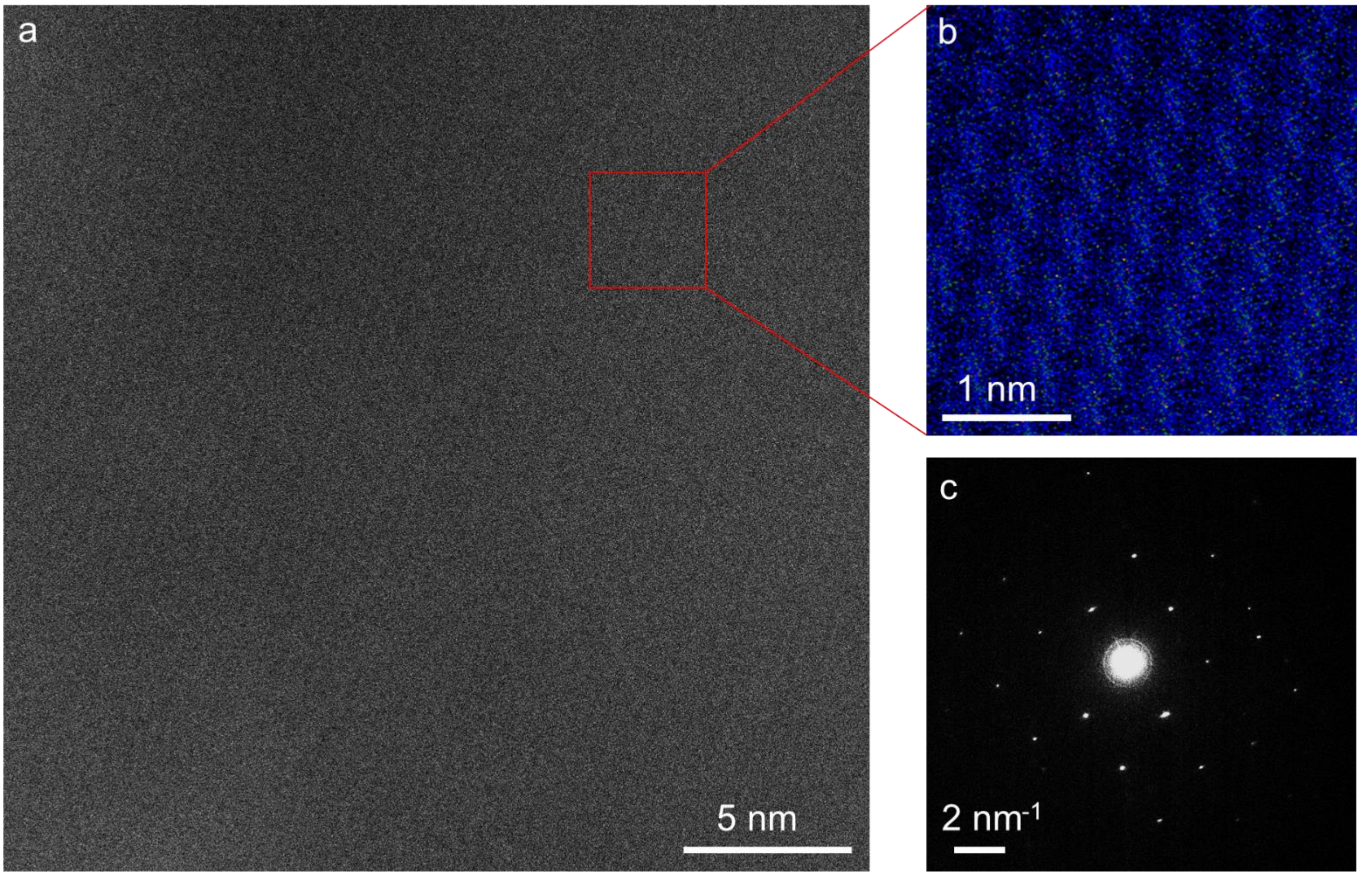

Supplementary Fig. 6. (a) HAADF-STEM image of 2D $PhenPtCl_2$ at 25 °C. (b) Enlarged HAADF-STEM image of 2D $PhenPtCl_2$ at 25 °C. (c) Selected area electron diffraction (SAED) of 2D $PhenPtCl_2$ at 25 °C. The crystal structure of 2D $PhenPtCl_2$ at 25 °C was characterized by AC-STEM. First, the atomic resolution HAADF-STEM image does not show the fine atomic structure of $PhenPtCl_2$, since the organic molecular structure of the $PhenPtCl_2$ crystal is sensitive to electron beam irradiation. However, the corresponding SAED patterns of 2D $PhenPtCl_2$ also indicate its single crystal structure.

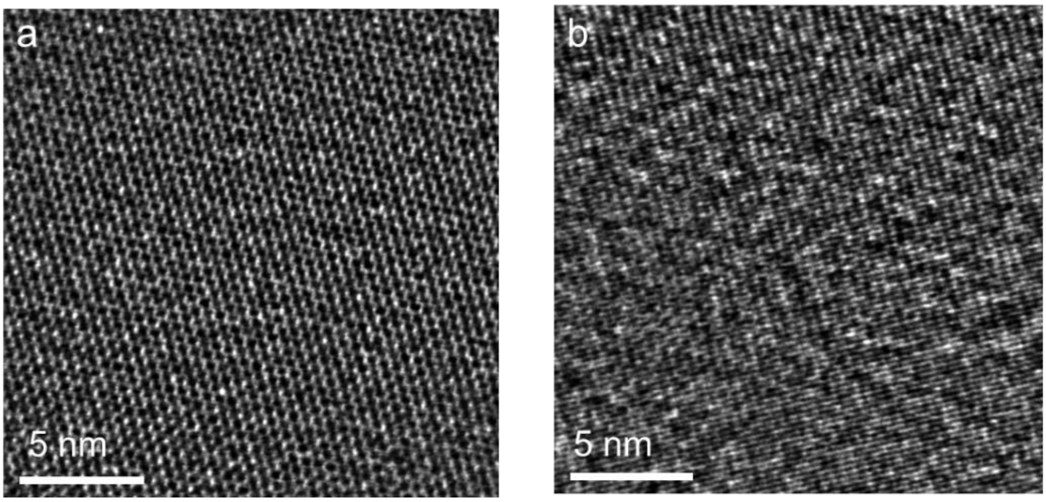

Supplementary Fig. 7. (a, b) Atomic-scale iDPC-STEM images of 2D $PhenPtCl_2$ at 25 °C from different zone axes. The iDPC-STEM images exhibit a clear and well-defined periodic arrangement in the crystal structure.

Supplementary Note 4: Growth mechanism of 2D $PhenPtCl_2$ nanosheets

The growth mechanism for 2D $PhenPtCl_2$ nanosheets was also investigated to study the relationship between the molar ratio of Pt to Phen in the precursor and the resulting thickness and morphology in Supplementary Fig. 8 - 10. When the molar ratio of Pt : Phen is 10:1, the 2D $PhenPtCl_2$ nanosheets reach a minimum thickness of 8.3 nm. As the ratio of Pt : Phen decreases, the thickness of 2D $PhenPtCl_2$ nanosheets gradually increases up to 177.3 nm (Supplementary Fig. 8), while the length/width ratio decreases from approximately 18 to around 1, and gradually increases by about 5. More optical images of 2D $PhenPtCl_2$ nanosheets at different molar ratio of Pt : Phen can be seen in Supplementary Fig. 9. These phenomena indicate that 2D Phen molecules play a crucial role in controlling the thickness of 2D $PhenPtCl_2$ nanosheets, and the inherent 2D properties of Phen molecule drive the growth along the 2D planes, in accordance with the layer growth theory of crystals. Moreover, the length/width ratio is also governed by the concentration of 2D Phen molecule, as the ultra-low concentration of Phen impels the growth of 2D $PhenPtCl_2$ nanosheets by the kinetic limiting mechanism. This results in rapid nanosheet growth in a specific direction, adhering to Bravais rule (Supplementary Fig. 10a). Furthermore, Raman characterization of 2D $PhenPtCl_2$ nanosheets with different thicknesses reveals a gradual increase in Raman peak intensity with thickness, accompanied by a slight blue shift, indicating improved crystal quality and increased nanosheet thickness of 2D $PhenPtCl_2$ nanosheets (Supplementary Fig. 10b).

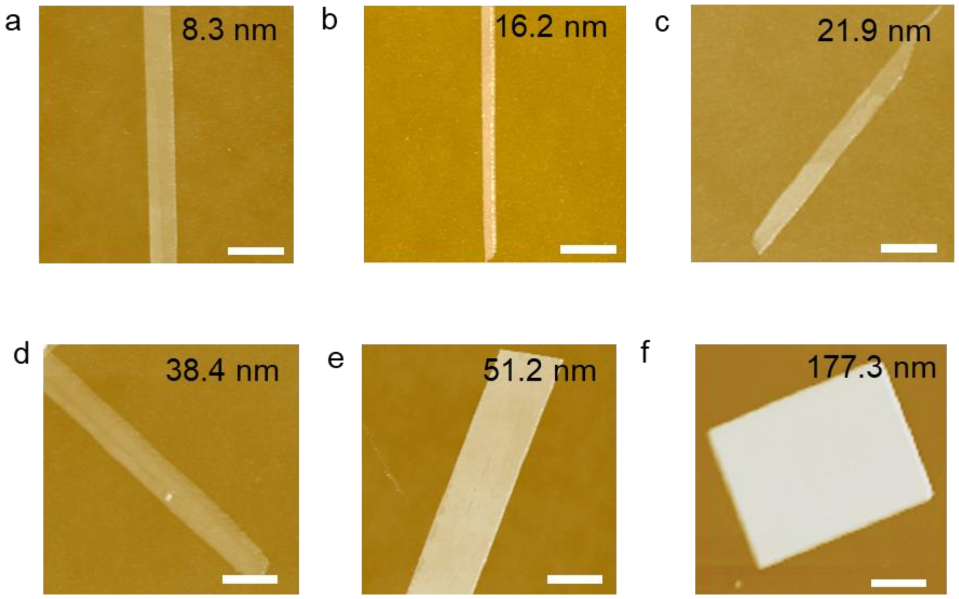

Supplementary Fig. 8. (a-f) Different thickness of 2D $PhenPtCl_2$ nanosheets from 8.3 nm to 177.3 nm.

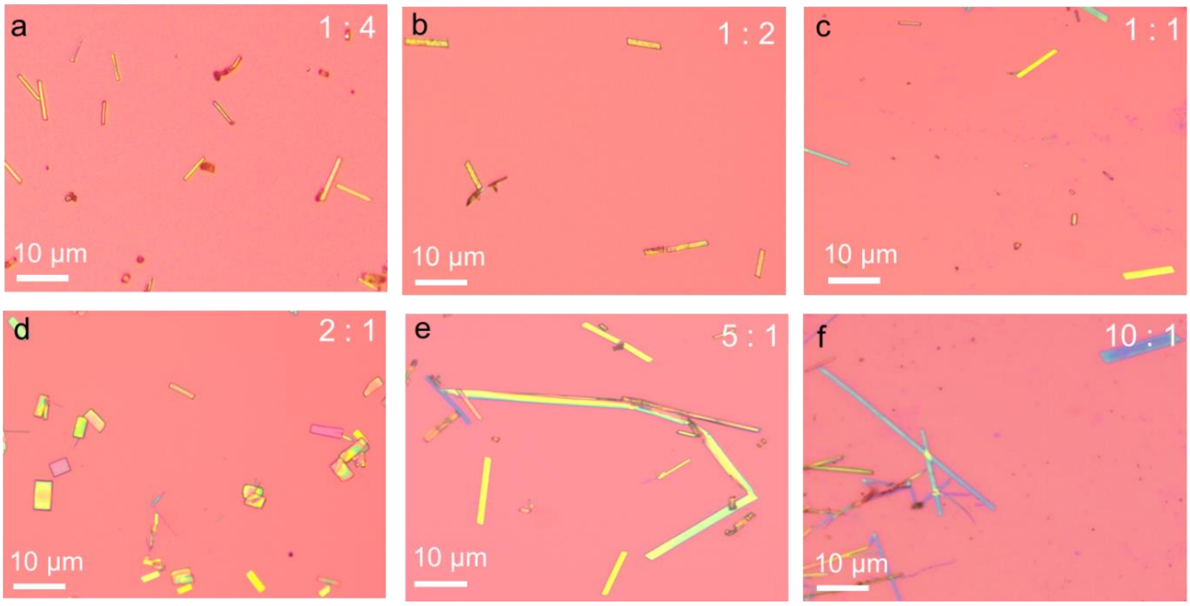

Supplementary Fig. 9. (a-f) Optical images of 2D $PhenPtCl_2$ nanosheets at different molar ratio of Pt : Phen. And molar ratio of Pt : Phen = 1:4, 1:2, 1:1, 2:1, 5:1, 10:1.

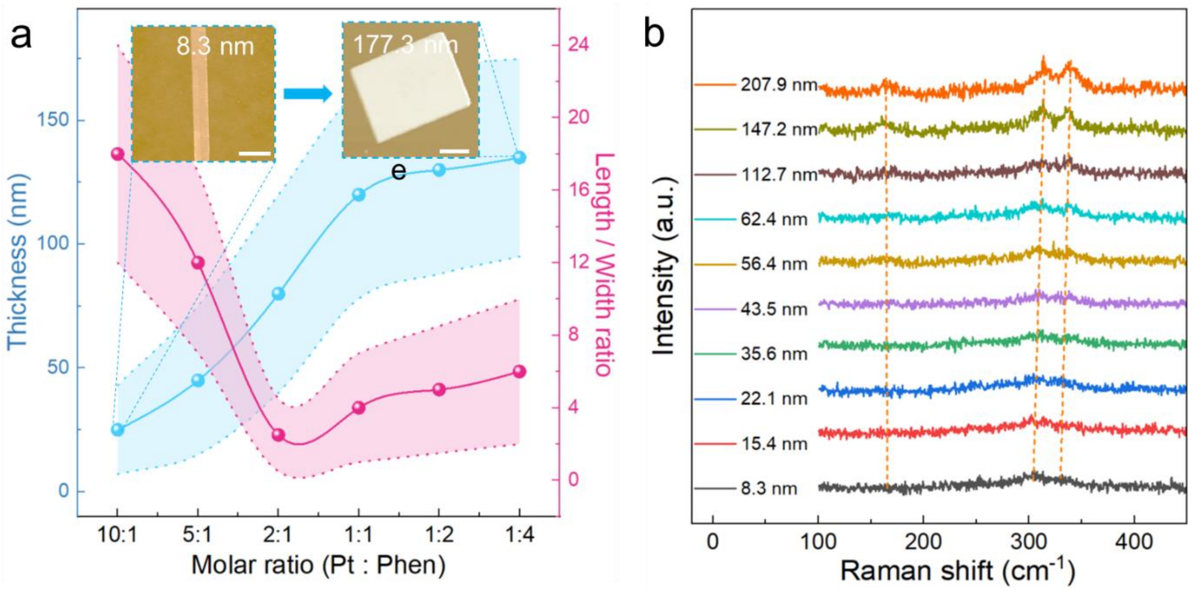

Supplementary Fig. 10. Structural evolution of the 2D $PhenPtCl_2$ crystal. (a) Thickness and length/width ratio trend changes with the Pt : Phen molar ratio. Inset: AFM image of 2D $PhenPtCl_2$ nanosheets with different thickness. (b) Raman properties of the 2D $PhenPtCl_2$ crystal with increasing thickness from 8.3 nm to 207.9 nm.

Supplementary Note 5: Structural analysis of 2D $PhenPtCl_2$ nanosheets under different annealing temperatures

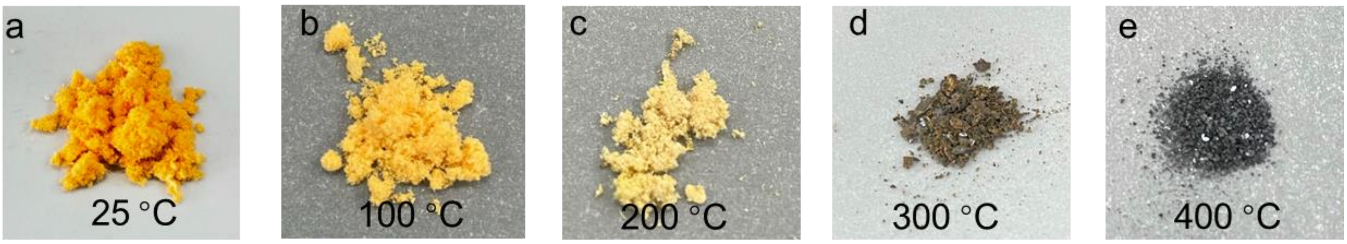

Supplementary Fig. 11. (a-e) Photographs of 2D $PhenPtCl_2$ organic crystals under different annealing temperatures from 25 °C to 400 °C. The color change of powder products changes from yellow to light yellow, and then dark brown to black.

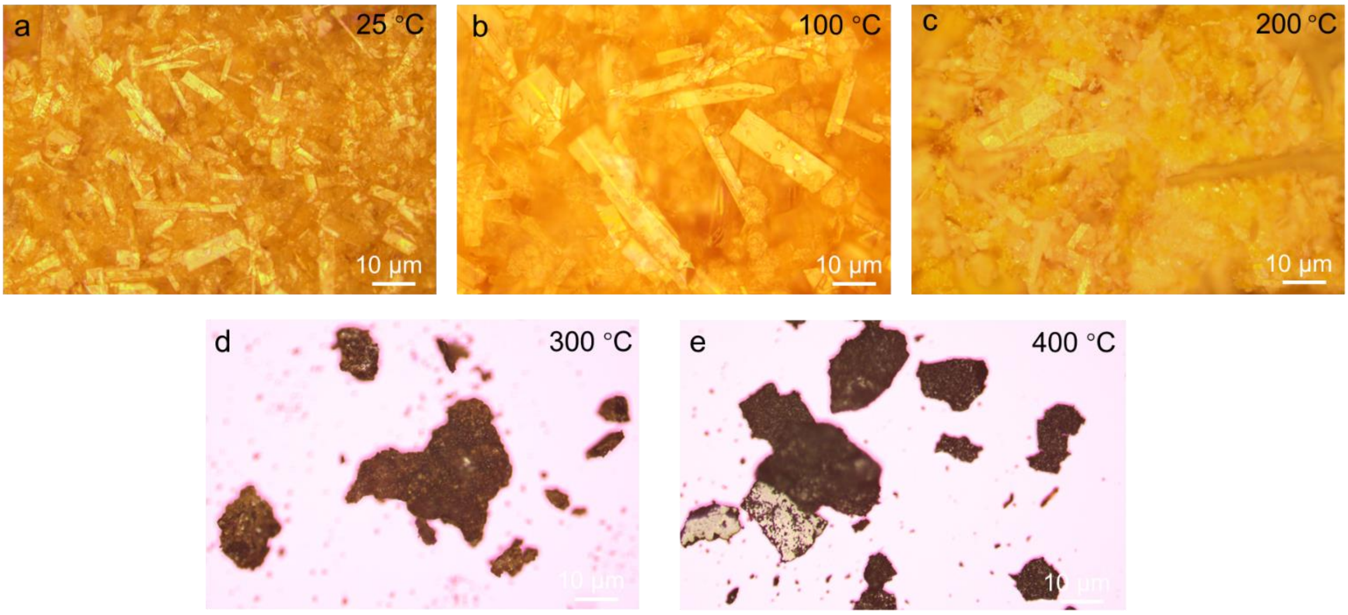

Supplementary Fig. 12. (a-e) Optical images of 2D $PhenPtCl_2$ organic crystals under different annealing temperatures from 25 °C to 400 °C. The optical images of the corresponding powder crystals show the lamellar structure. When the annealing temperature of 2D $PhenPtCl_2$ crystals reaches 200 °C, the crystal structure has a significant decomposition. As the annealing temperature of 2D $PhenPtCl_2$ crystals reaches 300 °C, the lamellar structure begins to collapse.

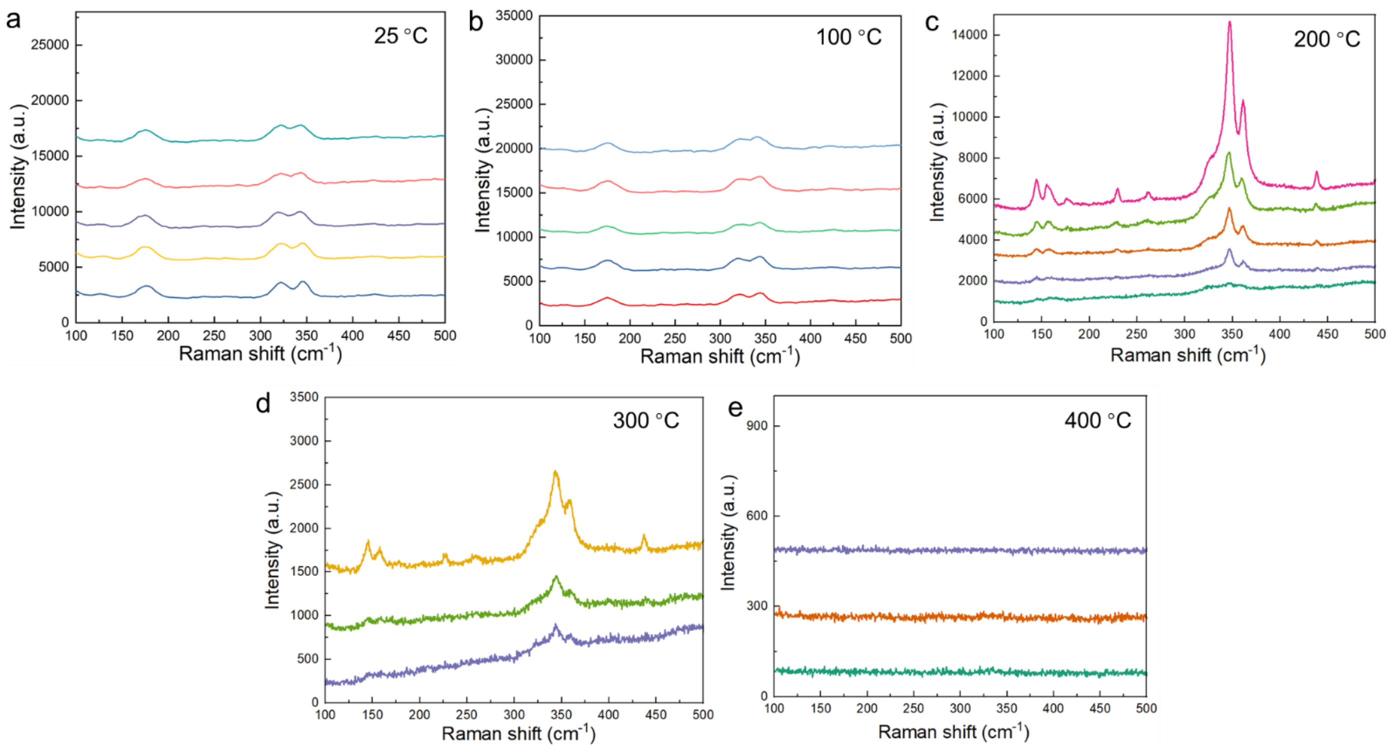

Supplementary Fig. 13. (a-e) Raman spectrum of the corresponding powder crystals from 25 °C to 400 °C. At 25 °C and 100 °C, the same Raman peak was observed. Whereas the Raman peaks at 200 °C and 300 °C show similarity but were distinct from those at 25 °C and 100 °C. Notably, no Raman peaks were detected at 400 °C, indicating that the $PhenPtCl_2$ crystal has already initiated decomposition, transforming into an amorphous carbon structure.

Supplementary Note 6: Atomic structural analysis of 2D $PhenPtCl_2$ nanosheets at 100 °C

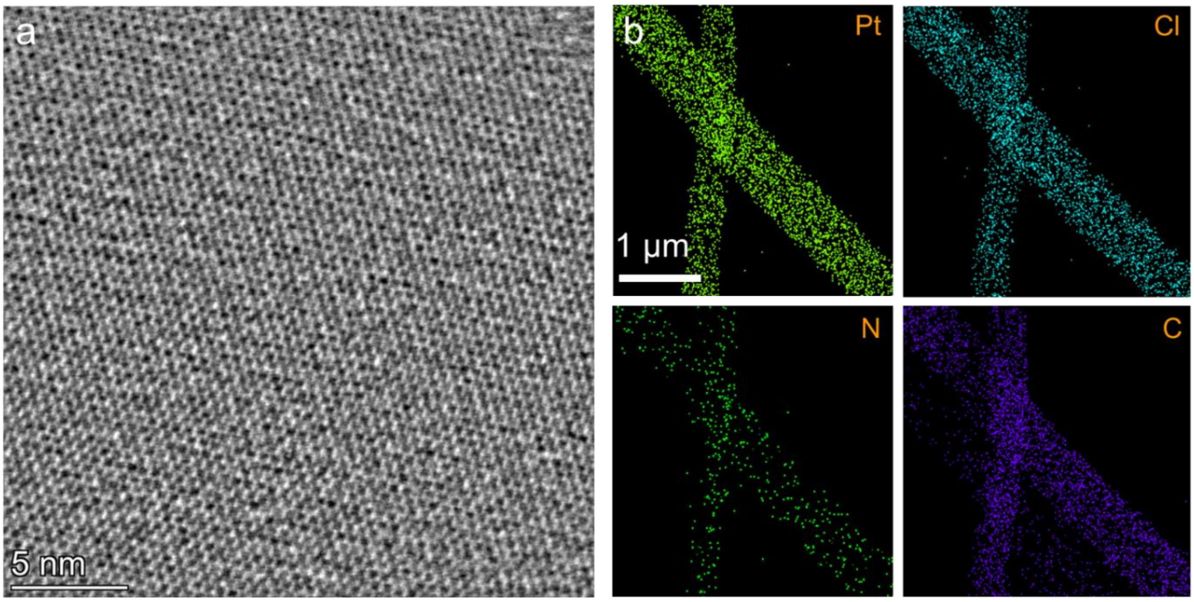

Supplementary Fig. 14. Atomic structural analysis of 2D $PhenPtCl_2$ nanosheets at 100 °C. (a) iDPC-STEM image and (b) EDS maps of 2D $PhenPtCl_2$ samples at 100 °C. iDPC-STEM imaging and EDS maps of 2D $PhenPtCl_2$ crystal at 100 °C confirm no obvious structural difference from the 2D $PhenPtCl_2$ crystal at 25 °C.

Supplementary Note 7: Structural stability of 2D $PhenPtCl_2$ nanosheets in the organic solvent

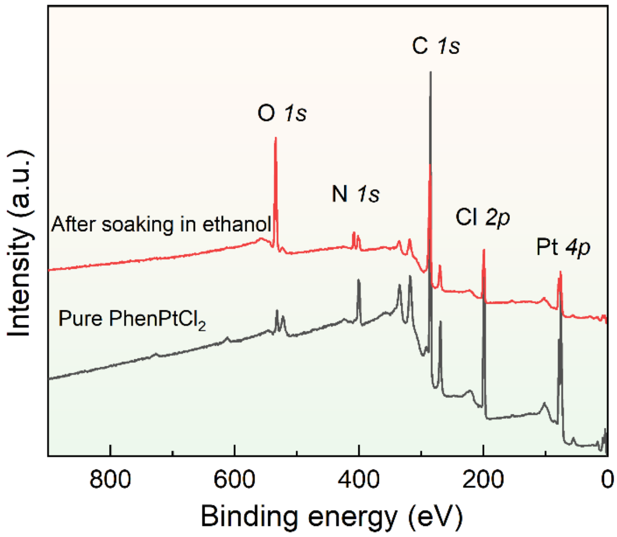

Supplementary Fig. 15. Overall XPS data of 2D $PhenPtCl_2$ samples compared with the XPS data after purification in ethanol for 8 h. There were no significant peak shifts and deletions of various elements.

Supplementary Note 8: XPS data of 2D PhenPtCl₂ nanosheets at 25 °C and 100 °C

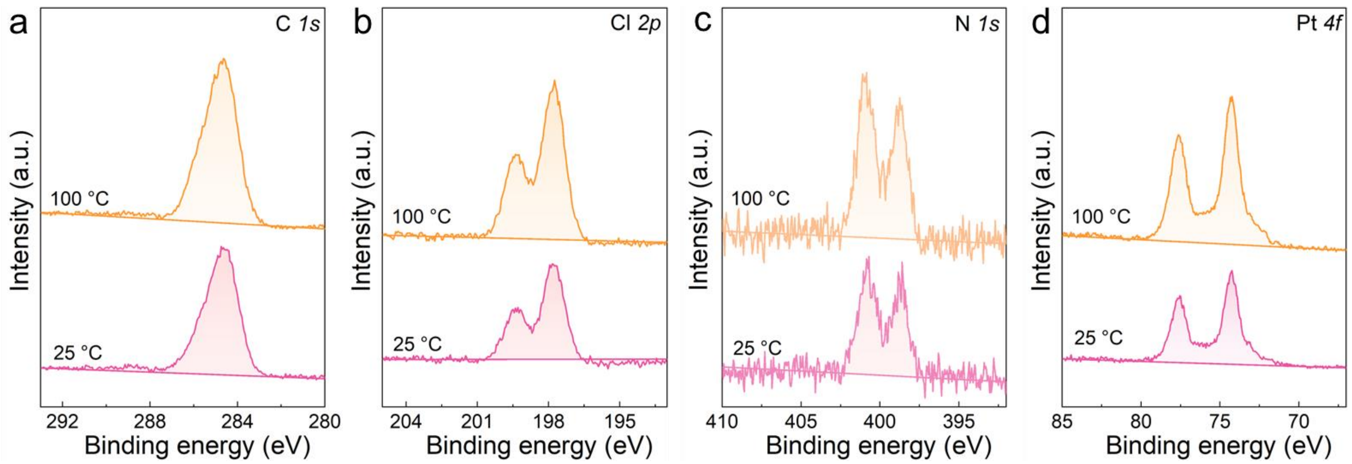

Supplementary Fig. 16. High-resolution XPS data of 2D $PhenPtCl_2$ crystal at 25 °C and 100 °C in (a) C *1s*, (b) Cl *2p*, (c) N *1s* and (d) Pt *4f*. Based on the XPS data of 2D $PhenPtCl_2$ samples at 25 °C and 100 °C, and no difference in valence state and peak position were revealed between 2D $PhenPtCl_2$ samples at 25 °C and 100 °C. So, it is the same 2D crystal at 25 °C and 100 °C, no crystal structure evolution occurred at 100 °C.

Supplementary Note 9: Structural analysis of 2D $PhenPtCl_2$ nanosheets at 100 °C

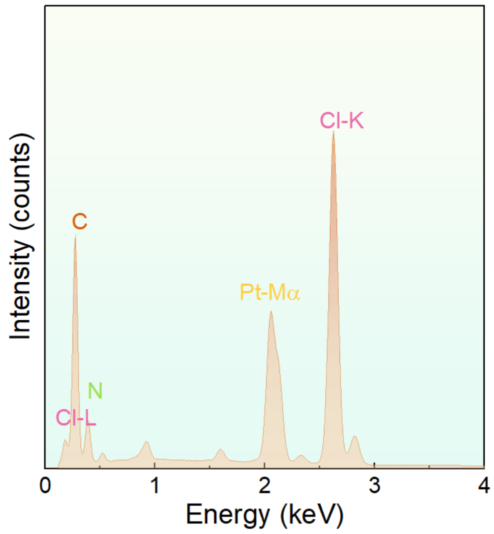

Supplementary Fig. 17. Energy dispersive spectroscopy of 2D $PhenPtCl_2$ nanosheets at 100 °C.

Supplementary Table 1. EXAFS fitting parameters at the Pt L_3-edge for 2D $PhenPtCl_2$ nanosheets at 100 °C.

Sample	Shell	CN	R(Å)	σ2(Å^2·10^{-3})	ΔE_0 (eV)	R factor (%)
$PhenPtCl_2$	Pt-N	2.3±0.2	1.98±0.022	0.0052	6.02±3.05	0.89
	Pt-Cl	2.0±0.3	2.29±0.034	0.0039	4.60±4.27	0.85

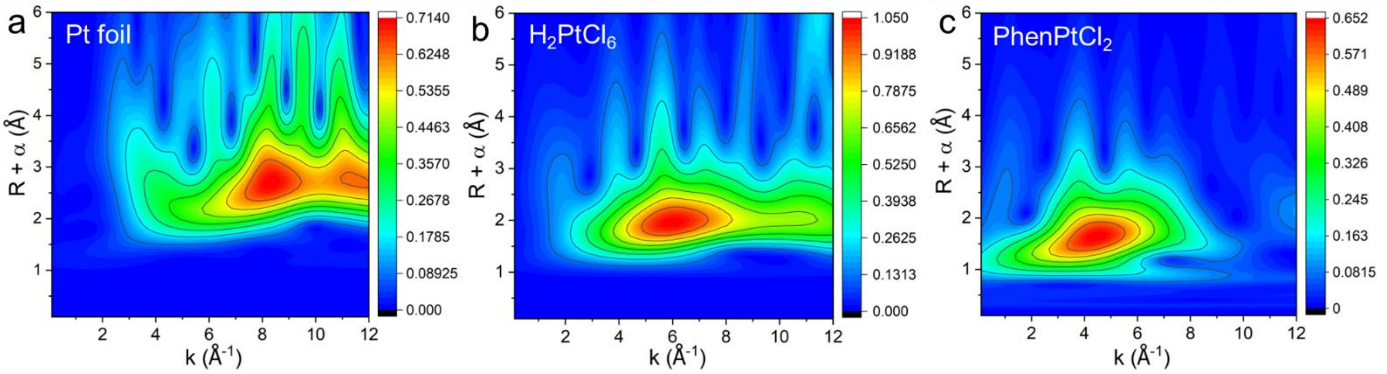

Supplementary Fig. 18. The WT for the Pt atom in EXAFS signals of (a) Pt foil, (b) H_2PtCl_6 powder and (c) 2D $PhenPtCl_2$ nanosheets at 100 °C.

Supplementary Note 10: HER evaluation of 2D PhenPtCl₂ nanosheets at different annealing temperatures

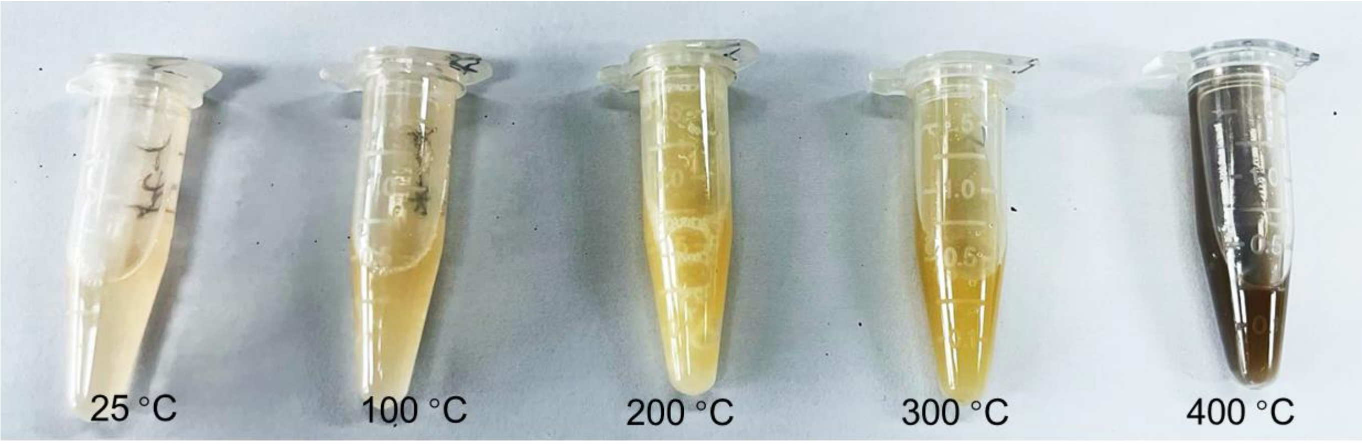

Supplementary Fig. 19. The corresponding inks of 2D $PhenPtCl_2$ nanosheets at different annealing temperatures from 25 °C to 400 °C.

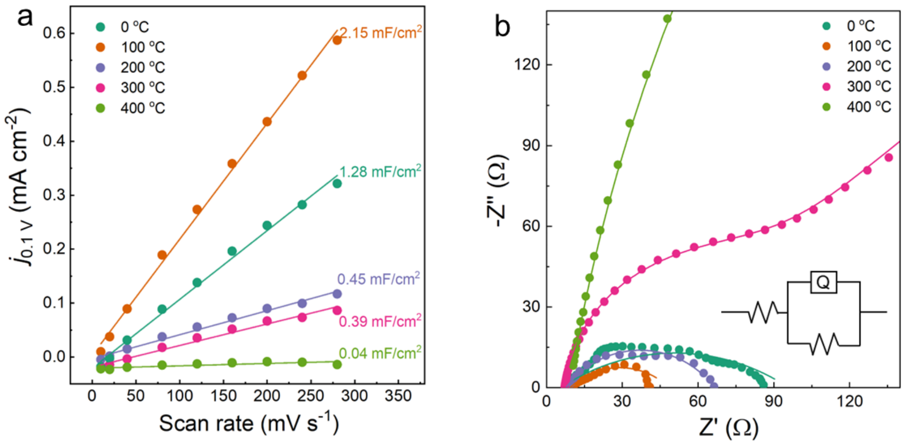

Supplementary Fig. 20. (a) The double-layer capacitance obtained from the slope of linear fitting (current density plotted as a function of scan rate) of 2D $PhenPtCl_2$ at 100 °C in acid solution. (b) Electrochemical impedance spectroscopy Nyquist plots of different catalysts. The 2D $PhenPtCl_2$ at 100 °C sample showed the largest electrochemical active surface area (ECSA) with 2.15 mF cm^{-2} and demonstrated the smallest transmission resistance with 32 Ω, which fully demonstrated the essential reasons for 2D $PhenPtCl_2$ at 100 °C sample to maintain excellent HER performance.

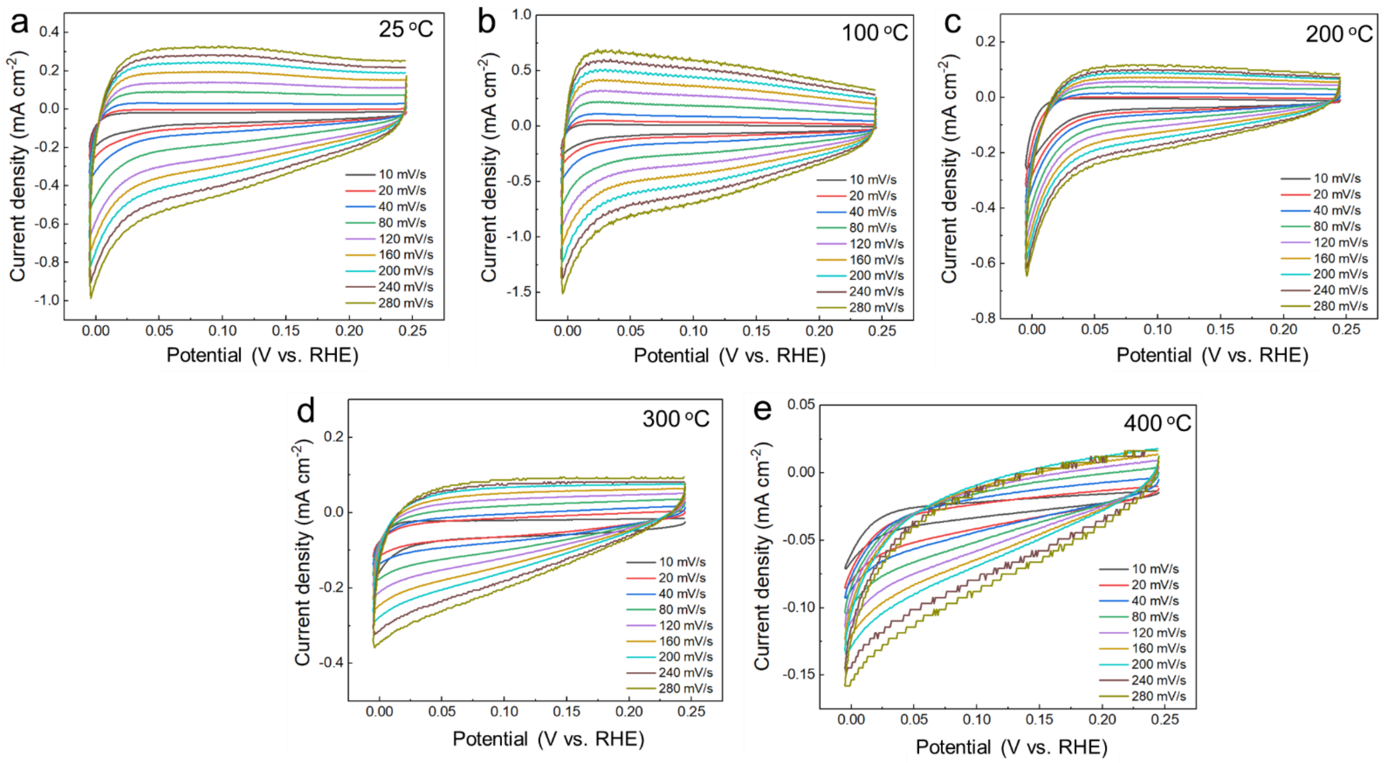

Supplementary Fig. 21. (a-e) Capacitive currents of 2D PhenPtCl$_2$ nanosheets at different annealing temperatures (25 °C, 100 °C, 200 °C, 300 °C and 400 °C) in acid solution, which can be measured from cyclic voltammetry in a potential range where non-Faradaic process is observed.

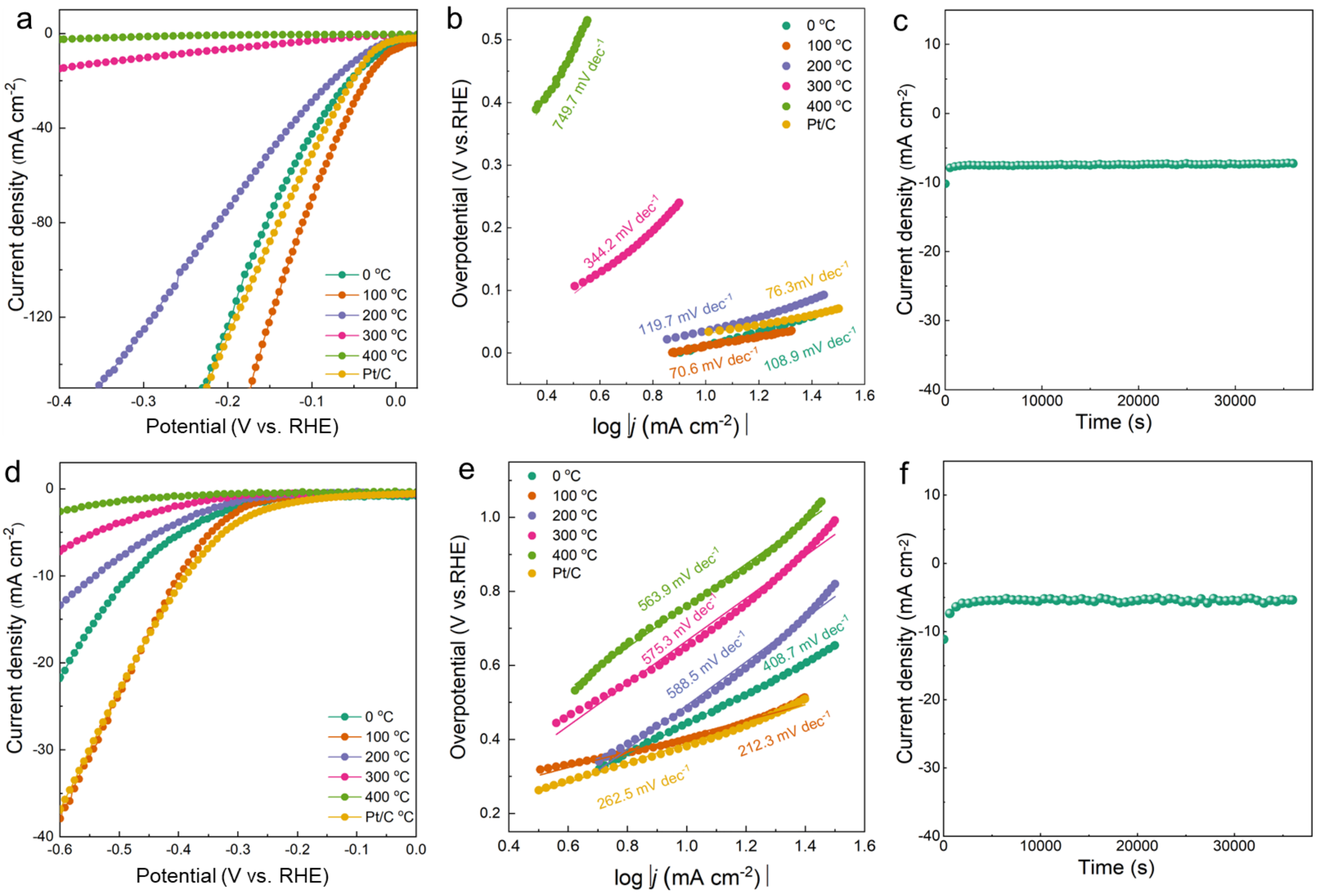

Supplementary Fig. 22. HER performance of 2D $PhenPtCl_2$ nanosheets at different annealing temperatures in neutral and alkaline solution. (a) Cathodic polarization curves of 2D $PhenPtCl_2$ nanosheets under different annealing temperatures in alkaline solution. (b) Corresponding Tafel slopes derived from polarization curves. (c) The durability test of 2D $PhenPtCl_2$ nanosheets at 10 mA cm^{-2} in alkaline solution. (d) Cathodic polarization curves of 2D $PhenPtCl_2$ nanosheets under different annealing temperatures in neutral solution. (e) Corresponding Tafel slopes derived from polarization curves. (f) The durability test of 2D $PhenPtCl_2$ nanosheets at 10 mA cm^{-2} in neutral solution.

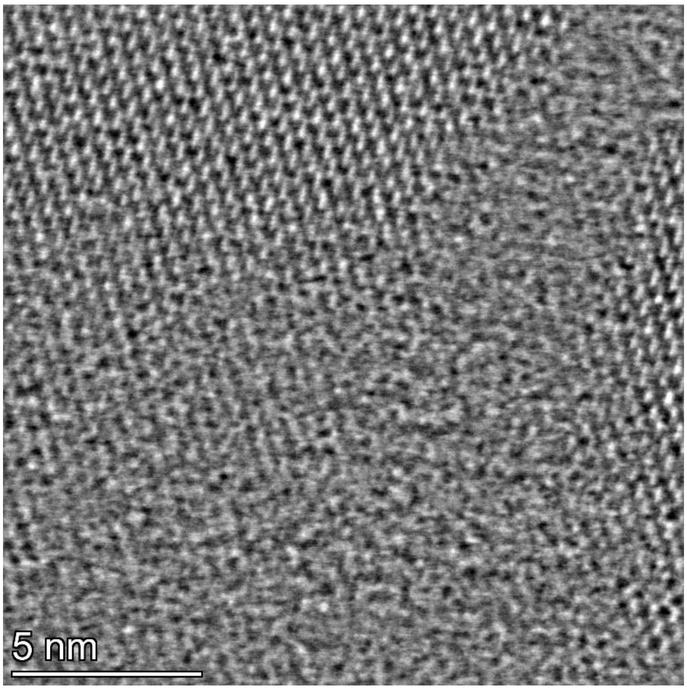

Supplementary Fig. 23. Atomic-scale iDPC-STEM image of 2D $PhenPtCl_2$ samples at 100 °C after the stability test end in 2 h. This atomic structure of 2D $PhenPtCl_2$ samples at 100 °C after the HER can be effectively proved, indicating that the crystal structure started to decompose partially but still maintains the original crystal cell structure with Phen-Pt-Cl_2.

Supplementary Note 11: Catalytic mechanism in structural evolution of 2D $PhenPtCl_2$ crystal

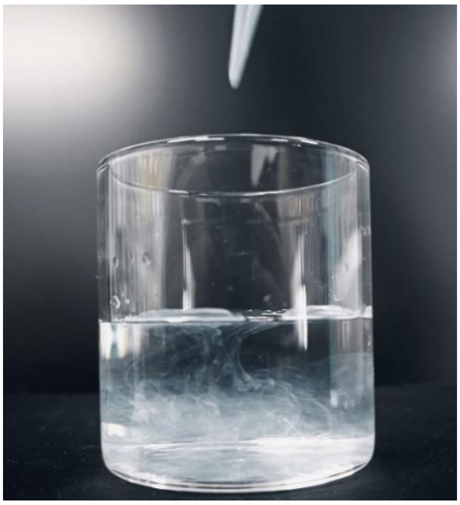

Supplementary Fig. 24. Optical image in the formation of numerous white precipitates in acid solution. In which, silver nitrate solution is employed to identify the presence of Cl^- ions in the electrolyte after electrocatalytic reaction.

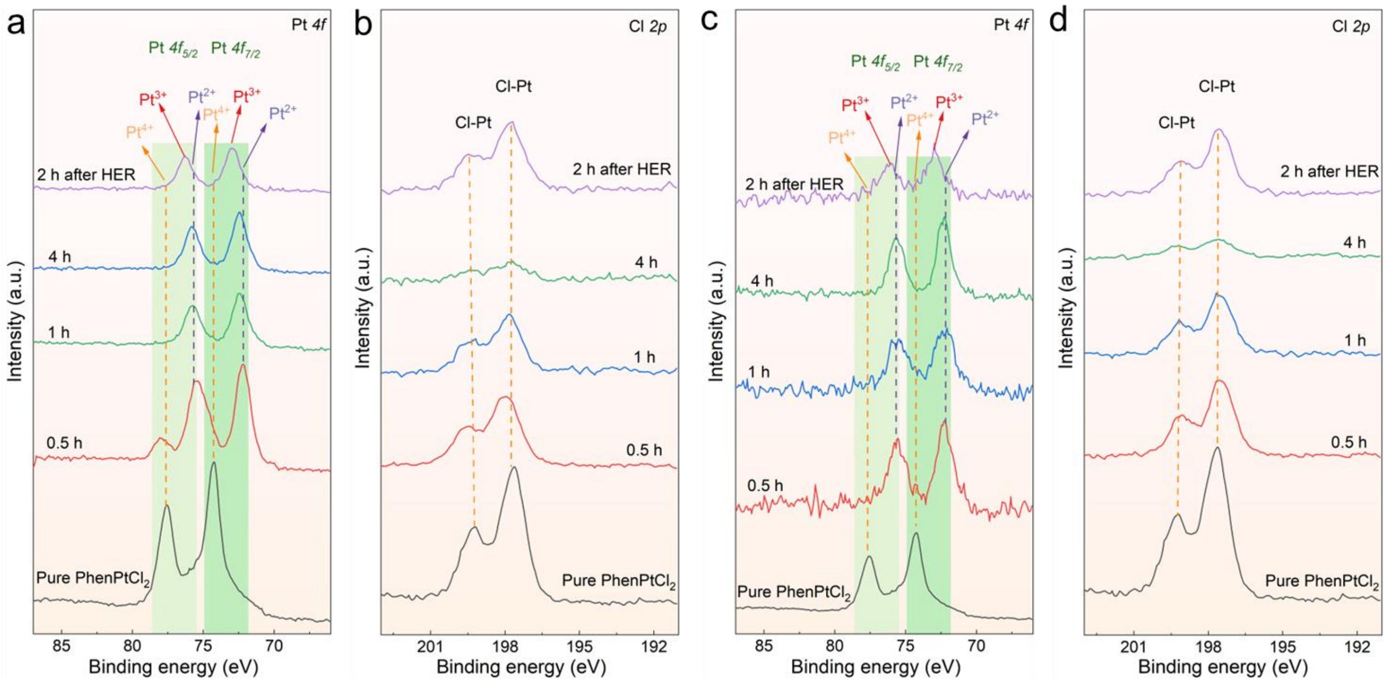

Supplementary Fig. 25. In-situ XPS data of 2D $PhenPtCl_2$ samples at 100 °C during the whole catalytic process in the neutral electrolyte and alkaline electrolyte. In-situ XPS data in (a) Pt *4f* peak and (b) Cl *2p* peak of 2D $PhenPtCl_2$ samples at 100 °C during the whole catalytic process and 2 h after the catalytic reaction in the neutral electrolyte. In-situ XPS data in (c) Pt *4f* peak and (d) Cl *2p* peak of 2D $PhenPtCl_2$ samples at 100 °C during the whole catalytic process and 2 h after the catalytic reaction in the alkaline electrolyte.

Supplementary Note 12: Computational details for catalytic mechanism of 2D $PhenPtCl_2$ crystal

Spin-polarized DFT calculations were performed by Vienna *ab initio* simulation package (VASP)[1]. The Perdew-Burke-Ernzerhof (PBE) exchange-correlation functional of generalized gradient approximation (GGA) was used[2]. To describe the van der Waals interaction, DFT-D3 method of Grimme et al. was employed[3,4]. For sampling the Brillouin zone, 2 × 2 × 1 k-points was adopted. The cutoff energy was set as 450 eV. The vacuum region of all the cells was not less than 15 Å above the plane.

The free energy calculation was based on computational hydrogen electrode (CHE) model to describe the ($H^+ + e^-$) transfer at pH = 0[5,6]. ΔG was calculated as $\Delta G = \Delta E - T\Delta S + \Delta ZPE$ (T = 298.15 K), where ΔE is the reaction energy obtained from DFT calculations. ΔS is entropy correction. ΔZPE is the correction in zero-point energy (ZPE). ΔS and ΔZPE were both calculated by VASPKIT code[7].

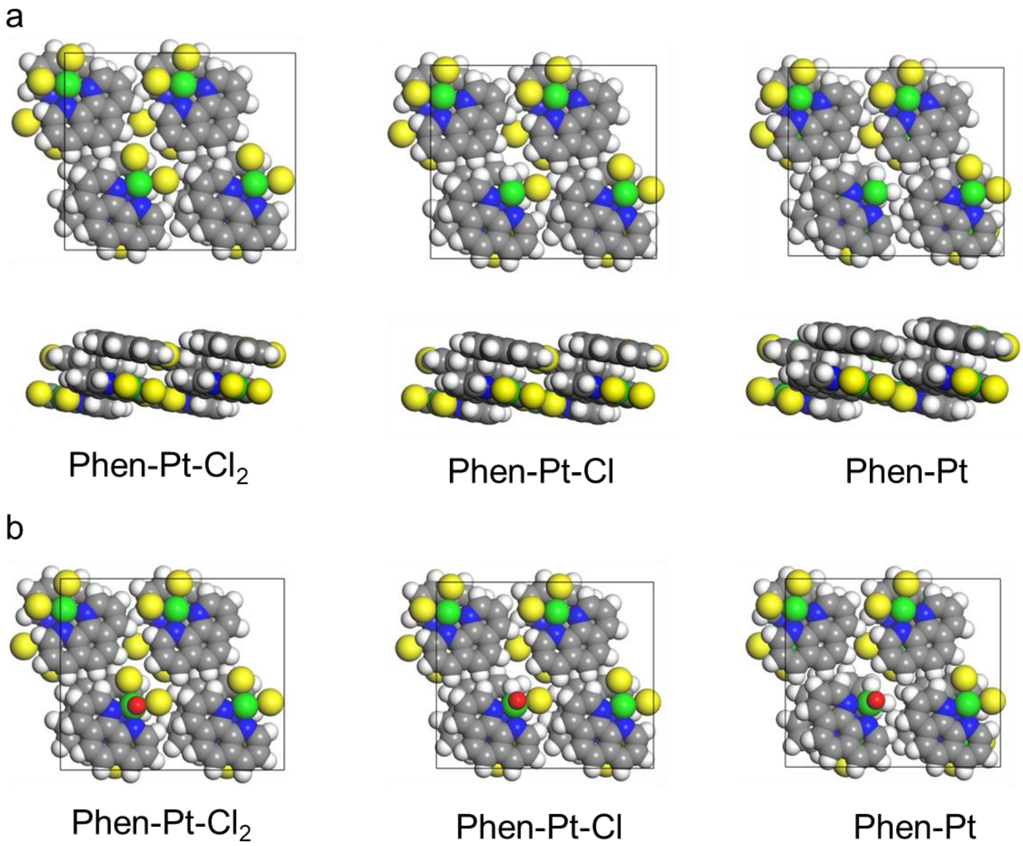

Supplementary Fig. 26. These models of HER adsorption. (a) Top and side views of Phen-Pt-Cl_2, Phen-Pt-Cl and Phen-Pt. (b) H adsorption on Phen-Pt-Cl_2, Phen-Pt-Cl and Phen-Pt. Green, yellow, grey, blue, and white balls represent Pt, Cl, C, N, H in the molecule, respectively. The red balls denote H adsorbed on the molecule.

References

1. Kresse G, Furthmuller J. Efficient iterative schemes for ab initio total-energy calculations using a plane-wave basis set. *Phys. Rev. B Condens. Matter.* **54**, 11169-11186 (1996).

2. Perdew JP, Burke K, Ernzerhof M. Generalized Gradient Approximation Made Simple. *Phys. Rev. Lett.* **78**, 1396-1396 (1997).

3. Grimme S, Antony J, Ehrlich S, Krieg H. A consistent and accurate ab initio parametrization of density functional dispersion correction (DFT-D) for the 94 elements H-Pu. *J. Chem. Phys.* **132**, 154104 (2010).

4. Grimme S, Ehrlich S, Goerigk L. Effect of the damping function in dispersion corrected density functional theory. *J. Comput. Chem.* **32**, 1456-1465 (2011).

5. Nørskov JK*, et al.* Origin of the Overpotential for Oxygen Reduction at a Fuel-Cell Cathode. *J. Phys. Chem. B* **108**, 17886-17892 (2004).

6. Peterson AA, Abild-Pedersen F, Studt F, Rossmeisl J, Nørskov JK. How copper catalyzes the electroreduction of carbon dioxide into hydrocarbon fuels. *Energy Environ. Sci.* **3**, 1311-1315 (2010).

7. Wang V, Xu N, Liu J-C, Tang G, Geng W-T. VASPKIT: A user-friendly interface facilitating high-throughput computing and analysis using VASP code. *Comput. Phys. Commun.* **267**, 108033 (2021).
